# Superficial capillary plexus vessel density/deep capillary plexus vessel density ratio in healthy eyes

**DOI:** 10.1186/s12886-022-02673-8

**Published:** 2022-12-09

**Authors:** Ki-Yup Nam, Min-Woo Lee, Kook-Hyung Lee, Jung-Yeul Kim

**Affiliations:** 1 Department of Ophthalmology, Chungnam National University Sejong Hospital, Daejeon, Republic of Korea; 2 Department of Ophthalmology, Konyang University College of Medicine, #1643 Gwanjeo-dong, Seo-gu, Daejeon, South Korea; 3 Department of Ophthalmology, Chungnam National University College of Medicine, #640 Daesa-dong, Jung-gu, Daejeon, 301-721 South Korea; 4 1.0 Eye clinic, Daejeon, Republic of Korea

**Keywords:** Optical coherence tomography angiography, Superficial capillary plexus, Deep capillary plexus, Vessel density

## Abstract

**Background:**

To identify factors differently affecting the superficial capillary plexus (SCP) and deep capillary plexus (DCP) in healthy eyes using their vessel density (VD) ratio.

**Methods:**

Healthy eyes were enrolled. The ratio between the VD of SCP and DCP (SVD/DVD ratio) was calculated. Pearson correlation analyses were performed to identify the relationships between this ratio and other factors.

**Results:**

The mean SVD and DVD were 36.2 ± 5.7 and 37.7 ± 4.9%, respectively, and the mean SVD/DVD ratio was 0.96 ± 0.15. The SVD was significantly correlated with the best-corrected visual acuity (BCVA) (*r* = − 0.368, *P* <  0.001), age (*r* = − 0.408, *P* <  0.001), and OCTA quality (*r* = 0.520, *P* <  0.001). The DVD was significantly correlated with the BCVA (*r* = − 0.150, *P* = 0.008), age (*r* = − 0.229, *P* <  0.001), and OCTA quality (*r* = 0.555, *P* <  0.001). Among various factors, age (*r* = − 0.296, P <  0.001), the BCVA (*r* = − 0.237, *P* <  0.001), axial length (*r* = 0.234, *P* <  0.001), and OCTA quality (*r* = 0.270, *P* < 0.001) were significantly correlated with the SVD/DVD ratio.

**Conclusions:**

Age, BCVA, axial length, and OCTA image quality were significantly correlated with the SVD/DVD ratio. Age, the BCVA, and OCTA quality were more strongly correlated with the SCP, and the axial length was more strongly correlated with the DCP.

## Background

Fluorescein angiography (FA) has been the gold standard for evaluation of the retinal microcirculation and microvasculature. However, FA is invasive and time-consuming, and it is difficult to observe the retinal microvasculature in detail because of dye diffusion. After the development of optical coherence tomography angiography (OCTA), which is non-invasive and enables visualization of retinal microvasculature with high resolution, OCTA has replaced FA in many clinical fields. One of the advantages of OCTA is that the retinal microvasculature can be examined in each retinal layer and can be quantified. OCTA yields various quantitative parameters including vessel density (VD), vessel length density, and the area of the foveal avascular zone (FAZ) of the superficial capillary plexus (from the internal limiting membrane [ILM] to the inner plexiform layer [IPL]; SCP) and the deep capillary plexus (from the outer border of the IPL to the outer border of the outer plexiform layer [OPL]; DCP) with relatively high reliability [1–4].

Previous studies have reported impairment of retinal microvasculature in various ophthalmic diseases using OCTA. Chen et al. [5] reported that changes in the fractal dimension in the DCP could be an early indicator of microvasculature changes associated with diabetic retinopathy. Another study found that VD of the DCP (DVD) decreased in abnormal multifocal electroretinography patients using hydroxychloroquine for rheumatoid arthritis, but VD in the SCP (SVD) did not [6]. Meanwhile, Chou et al. [7] reported a greater decrease in macular perfusion density of SCP than that of DCP in patients with non-arteritic anterior ischemic optic neuropathy. As such, the SCP and DCP can be differently affected by various conditions.

Therefore, we hypothesized that the factors affecting the normal retinal microvasculature would differ between the SCP and DCP. In this study, We derived the SVD/DVD ratio and identified factors that affect it.

## Methods

### Subjects

This retrospective, cross-sectional study adhered to the tenets of the Declaration of Helsinki and was approved by the Institutional Review Board of Konyang University Hospital, Daejeon, Korea. We reviewed the charts of patients who visited our retinal clinic for a floater, cataract, unilateral epiretinal membrane, macular hole, or intraocular lens dislocation, with fellow eyes without any ophthalmic disease. The inclusion criteria were age 30 years old or older, spherical equivalent within 6 diopters, no medial opacities that affect fundus imaging, and normal clinical ocular examination findings with no evidence of retinal pathologies. The retrospective review of participants’ records and the waiver of informed consent were approved by the Institutional Review Board/Ethics committee of Konyang University Hospital. We obtained patient information of best-corrected visual acuity (BCVA), intraocular pressure, spherical equivalent, axial length (using an IOL master; Carl Zeiss, Jena, Germany, version 5.02), and parameters of OCT and OCTA. The exclusion criteria consisted of a history of systemic disease including diabetes and hypertension, glaucoma, optic nerve disorder, intraocular pressure ≥ 21 mmHg, axial length ≥ 26 mm, intraocular surgery except for cataract extraction, and a BCVA < 20/40 (logMAR 0.301). If both eyes met the inclusion criteria, one eye was randomly selected.

### OCTA imaging protocol

**Fig. 1:**
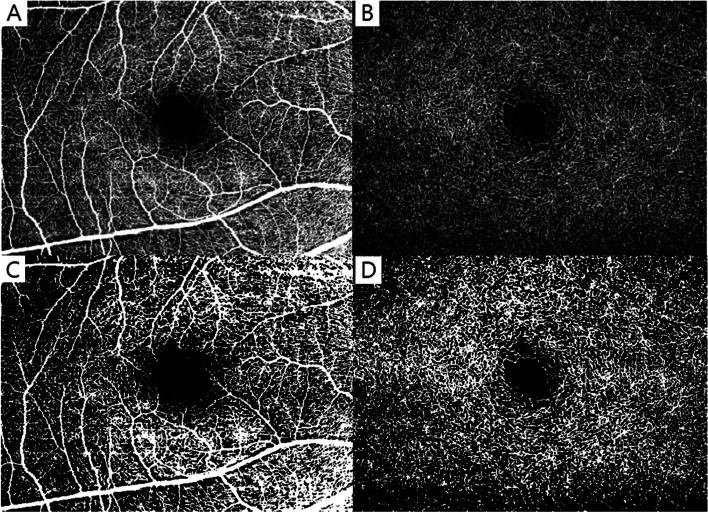
Original optical coherence tomography angiography images of the superficial capillary plexus (**A**) and deep capillary plexus (**B**), and images after conversion by ImageJ [(**C**) and (**D**), respectively]

OCTA was performed by a single skilled examiner using a Spectralis OCT2 device (Heidelberg Engineering, Heidelberg, Germany). The Spectralis OCT2 instrument can perform 70,000 A-scan/sec using a light source centered at 870 nm, with axial and transverse resolution of 3.0 and 6 μm in tissue, respectively. En face images of the SCP, defined as the layer from the ILM to the IPL, and the DCP, defined as the layer from the outer border of the IPL to the OPL, were visualized automatically by segmenting two separate slabs defined by arbitrary segmentation lines, which are created by the device software. In this study, a full area of 6.0 × 4.5 mm OCTA scans centered on the fovea was acquired and analyzed. The VD was calculated as the percentage area occupied by blood vessels, with the blood vessels being defined as pixels having decorrelation values above the threshold level using ImageJ software (National Institutes of Health, Bethesda, MD, USA )[8, 9]. After setting images with 8-bit type, the adjusted threshold tool was applied with default settings, and the dark background option was selected as a previous study [10]. This tool automatically sets the lower and upper threshold values (arbitrarily chosen as 110 and 255, respectively, for every image) and segmented grayscale images into features of interest and background (Fig. 1). The Littman and Bennett formulas were used to correct the magnification effect and to calculate true image siz e[11, 12]. In short, the relationship between the measured OCTA image diameter (Dm) and the true diameter of the fundus (Dt) was computed using the equation: Dt = p × q × Dm; where p × q was the overall image magnification factor, p was the magnification factor of the imaging system and q was that of the eye. Factor q was determined from the Bennett formula: q = 0.01306 × (axial length – 1.82), where 1.82 is a constant related to the distance between the corneal apex and the second principal plane. Factor p was computed from the Bennett formula if the axial length at which Dt = Dm is known [13]. For the Heidelberg systems used, the value of the magnification factor (p) was 3.39, given a nominal axial length of 24.385 mm. The OCTA quality is expressed in dB and ranges from 0 to 40. Any image exhibiting fixation loss, segmentation errors, motion artifacts, or the value of OCTA quality < 25 dB was excluded.

### Statistical analysis

Mean and standard deviation values were calculated for continuous variables, and frequency and percentage were calculated for categorical variables. All statistical analyses were performed using SPSS statistical software (version 18.0; IBM Corp., Armonk, NY, USA). Pearson correlation analyses were performed to identify the relationship between the SVD/DVD ratio and variable factors.

## Results

### Demographics and the SVD and the DVD values

A total of 222 eyes were enrolled. The demographic characteristics including age, sex, BCVA, axial length, or central macular thickness are listed in Table 1.

**Table 1:** Demographics and characteristics of participants

Number of cases	222
Age (mean ± SD, years)	57.5 ± 13.5
Sex (male, %)	115 (51.8)
Laterality (right, %)	114 (51.4)
Lens status (phakic, %)	207 (93.2)
BCVA (mean ± SD, logMAR)	0.05 ± 0.09
Spherical equivalent (mean ± SD, diopter)	−0.49 ± 2.63
Intraocular pressure (mean ± SD, mmHg)	13.1 ± 2.8
Axial length (mean ± SD, mm)	23.9 ± 1.0
Central macular thickness (mean ± SD, μm)	266.9 ± 23.5

*BCVA* best-corrected visual acuity

The mean SVD was 36.2 ± 5.7%, the mean DVD was 37.7 ± 4.9%, and the SVD/DVD ratio was 0.96 ± 0.15 (Table 2).

**Table 2:** Optical coherence tomography angiography parameters of participants

SVD (mean ± SD, %)	36.2 ± 5.7
DVD (mean ± SD, %)	37.7 ± 4.9
OCTA quality (mean ± SD,dB)	32.7 ± 3.0
SVD/DVD ratio (mean ± SD)	0.96 ± 0.15

*SVD* vessel density of the superficial capillary plexus; *DVD* vessel density of the deep capillary plexus; *OCTA* optical coherence tomography angiography

### Correlations between OCTA parameters and various factors

The SVD was negatively correlated with the BCVA (*r* = − 0.368, *P* < 0.001) and age (*r* = − 0.408, *P* < 0.001), and positively correlated with OCTA quality (*r* = 0.520, *P* < 0.001). The axial length was not significantly associated with the SVD (*r* = − 0.227, *P* = 0.374). The DVD was negatively correlated with BCVA (*r* = − 0.150, *P* = 0.008) and age (*r* = − 0.299, *P* < 0.001), positively correlated with OCTA quality (*r* = 0.555, *P* < 0.001). The axial length was significantly correlated with the DVD, which was different from the SVD (*r* = − 0.307, *P* = 0.014).

**Fig. 2:**
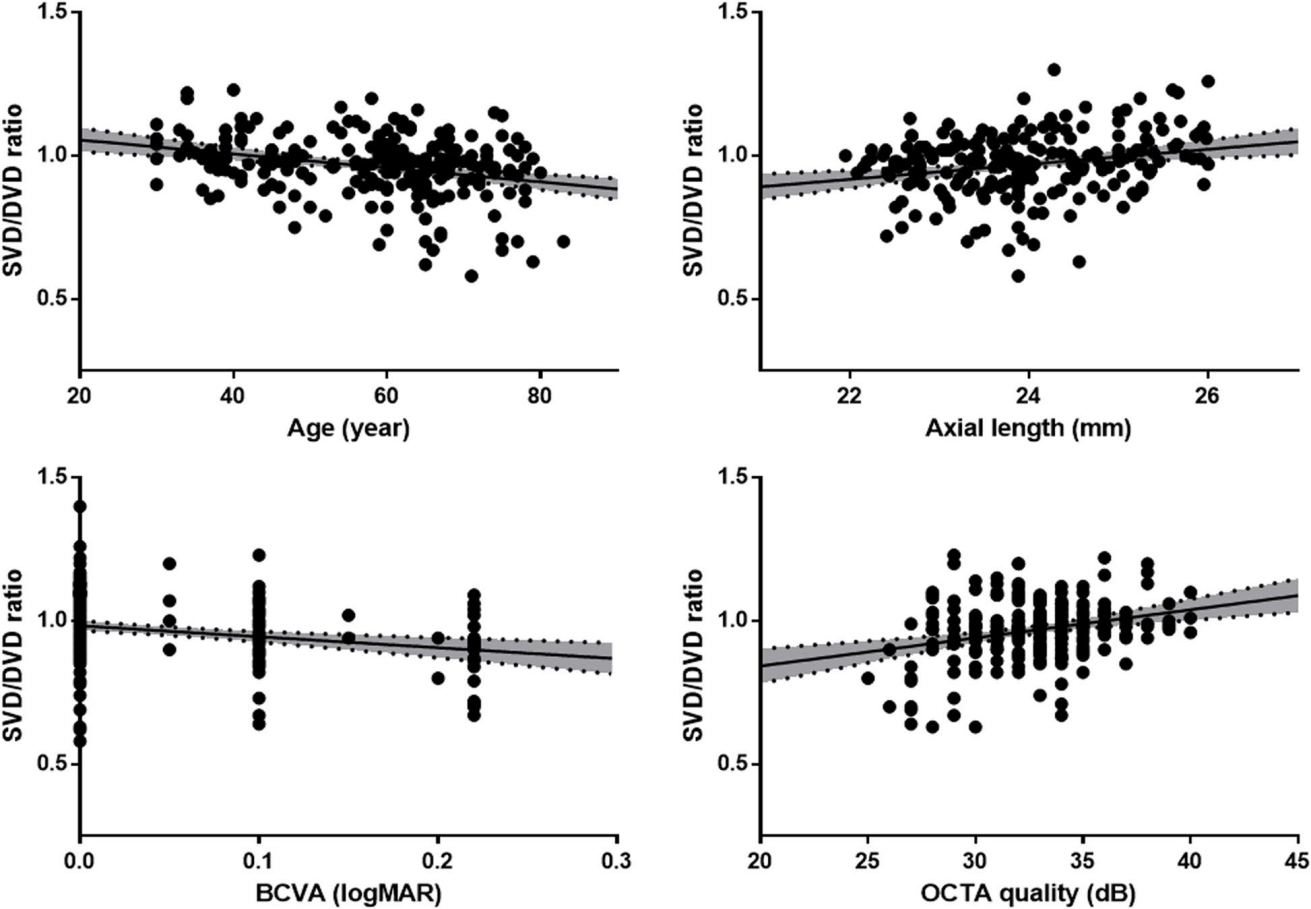
Scatter plots showing a significant correlation between the SVD/DVD ratio and age (*P* < 0.001), the BCVA (*P* < 0.001), axial length (*P* = 0.002), and OCTA quality (*P* < 0.001)

Among various factors, age (*P* < 0.001) and the BCVA (*P* < 0.001) were negatively correlated, and the axial length (*P* < 0.001) and OCTA quality (*P* < 0.001) were positively correlated with the SVD/DVD ratio (Table 3 and Fig. 2).

**Table 3:** Correlations between the SVD/DVD ratio and other factors

	Coefficient	*P* value
Age	−0.296	**< 0.001**
Sex	−0.160	0.620
Laterality	0.040	0.980
BCVA	−0.237	**< 0.001**
Spherical equivalent	−0.204	**0.021**
Intraocular pressure	0.102	0.350
Lens status	−0.135	0.250
Axial length	0.234	**< 0.001**
Central macular thickness	0.201	0.088
OCTA quality	0.270	**< 0.001**

*BCVA* best-corrected visual acuity; *OCTA* optical coherence tomography angiography

## Discussion

In an OCTA image, the SCP appears as a defined silhouette morphology with a linear and continuous white shape against a black background, which is lying in the retinal nerve fiber layer (RNFL) and the ganglion cell layer (GCL). The DCP is shown as a regular distribution around the FAZ with many complex, tiny radial and horizontal interconnections lying in the boundary plane between the inner nuclear layer (INL) and the OPL [14]. These differences in SCP and DCP locations, sizes, and morphologies suggest that various factors might affect them differently [9]. We tried to analyze this using the SVD/DVD ratio in normal eyes and found that age, the BCVA, the axial length, and OCTA quality were significantly correlated with the SVD/DVD ratio. However, since the SVD and DVD values are different for each OCTA device, different OCTA devices may show different SVD/DVD ratios from our study, which would give somewhat different results from ours statistically [15, 16]. However, the trend is expected to remain the same and we believe that our findings would be helpful for clinical situations and research using OCTA.

Previous studies reported a retinal microvascular decrease in eyes with high myopia, which would result from the elongation of eyeballs [17–19]. We found that the axial length was positively correlated with the SVD/DVD ratio, which could mean that the longer the axial length, the greater the DVD reduction than the SVD reduction. Actually, the axial length was significantly correlated with only the DVD, not the SVD in our study. You et al. [9] reported that longer axial length was significantly associated with the lower DVD, not with the lower SVD in their multivariate analyses, which is consistent with our study. This result would be associated with a previous study about the retinal layer thickness of high myopia. Kim et al. [20] reported that in high myopia, both the inner and outer rings of the ETDRS grid were thinned in the INL (where the DCP is located). However, only the outer ring was thinned in the inner retinal layer including the GCL and IPL (where the SCP is located), indicating that the range of the thinned INL was wider than that of the thinned inner retinal layer. The density of the smaller vessels in the DCP is greater than that in the SCP, and these smaller vessels may be more sensitive to elongation change, potentially leading to a greater decrease of the DVD than SVD according to increasing axial length [14, 21]. However, this result associated with axial length would contain a limitation that we excluded the eyes with axial length over 26.0 mm. Further studies including more range of axial length including ≥26.0 mm are needed to confirm this hypothesis.

When analyzing OCTA images, OCTA quality is crucial, which ranges from 0 (no signal) to 40 dB (excellent quality) and is considered good if the value is over 25 dB. Similar concepts are signal strength or signal strength index in other devices. Previous studies found that these values affected the measurement of VD and its reliability [1, 2, 9, 22–24]. You et al. [9] reported that a lower SVD and DVD were significantly associated with a lower signal strength index, and it was the strongest impact on the measured retinal VD. Additionally, even if the quality value is relatively high so the reliability of the test is good, such value can still affect the OCTA parameters. Lim et al. [25] reported that as the signal strength increased from 7 to 10, the OCTA parameters including VD and perfusion density increased. Our study also found that the SVD/DVD ratio was positively correlated with OCTA quality in images with over 25 dB, indicating that the SVD is more directly affected by OCTA quality than the DVD. Although the exact mechanism is unclear, OCTA quality should be considered when comparing retinal VDs, especially of SCP.

Yu et al. [22] found that the parafoveal flow index and vessel area density decrease with increasing age at a rate of 0.6 and 0.4% per year. Jo et al. [8] also reported that age substantially affected VD in most of the peripapillary and macular areas. In our study, age was negatively correlated with SVD and DVD, which is consistent with previous studies. Additionally, the SVD/DVD ratio was significantly correlated with age. This would result from different locations of the SCP and DCP. The thickness of the inner retina including the RNFL, GCL, and IPL, which locates SCP, is known to decrease with increasing age. Previous studies reported a decrease in the RNFL and GC-IPL with increasing age not only in diseased eyes but also in healthy eyes [26–31]. Additionally, the close relationship between the inner retinal layer thickness and the SVD has been known through many studies [32–35]. Therefore, the SVD may interact with a thinning inner retina over time and decrease along with them. Whereas, the DCP lies on the INL and its thickness is relatively less affected by age. Therefore, the SCP would be more sensitive to VD changes with aging than the DCP.

You et al. [9] reported that lower SVD and DVD were significantly associated with worse BCVA in a normal population in their univariate linear regression analysis. Our study showed a significant correlation between the BCVA and both the SVD and DVD in healthy eyes, which is consistent with a previous study. We also found that the SVD/DVD ratio was negatively correlated with the BCVA. The SVD located in the inner retina, which could be sensitive to ischemia associated with the reduction in macular perfusion by the aging effect and subsequently contributed to the photoreceptor cell damage and impaired BCVA, seemed to be correlated with BCVA more directly than the DVD in healthy eyes [8, 9, 22]. However, since some eyes with relatively poor visual acuity were included due to low BCVA criteria in the study, we could not totally exclude the possibility that a few eyes would have contained ophthalmic disease potentially that was not discovered by the examinations performed at that time. Further studies are needed to confirm this result.

Our study has several limitations. First, the retrospective nature of the work inevitably introduces some selection bias. Second, since eyes with axial length ≥ 26.0 mm were excluded, additional studies including high myopia are needed to more accurately identify the relationship between axial length and the OCTA parameters as we mentioned above. Third, the OCTA measurements were not performed at the same period of the day, which may cause diurnal variation. However, a previous study reported that the macular VD showed no significant diurnal variation, unlike the peripapillary VD [36]. So diurnal variation would not have a decisive effect on the results of our study. Fourth, we could not analyze the patient’s body mass index or smoking status, which could also affect macular VD, because of lacking relevant information on the chart [37, 38]. The strength of this study was that we included a relatively large number of patients from a wide range of age groups, and enrolled OCTA images with OCTA quality ≥25 dB, allowing accurate analyses. Additionally, this is the first study to identify different impacts on the SCP and DCP by various factors via SVD/DVD ratio.

## Conclusions

We investigated through the SVD/DVD ratio that various factors can affect SCP and DCP differently in healthy eyes and found that age, BCVA, axial length, and OCTA quality of OCTA images were significantly correlated with the SVD/DVD ratio. From these results, we were able to infer that age, BCVA, and OCTA quality were more correlated with the SCP, while the axial length was more correlated with the DCP. Physicians should consider these results when interpreting OCTA images. Our results may also facilitate future OCTA analyses of retinal microvasculature according to the retinal layer in eyes with various diseases.

## Data Availability

The datasets generated during and/or analyzed during the current study are available from the corresponding author on reasonable request.

## Abbreviations

FA: Fluorescein angiography
OCTA: Optical coherence tomography angiography
VD: Vessel density
FAZ: Foveal avascular zone
ILM: Internal limiting membrane
IPL: Inner plexiform layer
SCP: Superficial capillary plexus
OPL: Outer plexiform layer
DCP: Deep capillary plexus
DVD: Vessel density of the deep capillary plexus
SVD: Vessel density of superficial capillary plexus
BCVA: Best-corrected visual acuity
RNFL: Retinal nerve fiber layer
GCL: Ganglion cell layer
INL: Inner nuclear layer
